# Delivering Crocetin across the Blood-Brain Barrier by Using γ-Cyclodextrin to Treat Alzheimer’s Disease

**DOI:** 10.1038/s41598-020-60293-y

**Published:** 2020-02-27

**Authors:** Ka Hong Wong, Yuning Xie, Xiao Huang, Kazunori Kadota, Xin-Sheng Yao, Yang Yu, Xiaoyu Chen, Aiping Lu, Zhijun Yang

**Affiliations:** 1 School of Chinese Medicine, Hong Kong Baptist University, 7 Baptist University Road, Kowloon Tong, Kowloon Hong Kong; 2 Department of Formulation Design and Pharmaceutical Technology, Osaka University of Pharmaceutical Sciences, 4-20-1 Nasahara, Takatsuki, Osaka 569-1094 Japan; 3 Institute of Traditional Chinese Medicine & Natural Products, College of Pharmacy and Guangdong Province Key Laboratory of Pharmacodynamic Constituents of TCM and New Drug Research, Jinan University, Guangzhou, 510632 China; 4 Changshu Research Institute, Hong Kong Baptist University, Changshu Economic and Technological Development (CETD) Zone, Changshu, 215500 China

**Keywords:** Drug delivery, Drug delivery

## Abstract

Crocetin (CRT) has shown various neuroprotective effects such as antioxidant activities and the inhibition of amyloid β fibril formation, and thus is a potential therapeutic candidate for Alzheimer’s disease (AD). However, poor water solubility and bioavailability are the major obstacles in formulation development and pharmaceutical applications of CRT. In this study, a novel water-soluble CRT-γ-cyclodextrin inclusion complex suitable for intravenous injection was developed. The inclusion complex was nontoxic to normal neuroblastoma cells (N2a cells and SH-SY5Y cells) and AD model cells (7PA2 cells). Furthermore, it showed stronger ability to downregulate the expression of C-terminus fragments and level of amyloid β in 7PA2 cell line as compared to the CRT free drug. Both inclusion complex and CRT were able to prevent SH-SY5Y cell death from H_2_O_2_-induced toxicity. The pharmacokinetics and biodistribution studies showed that CRT-γ-cyclodextrin inclusion complex significantly increased the bioavailability of CRT and facilitated CRT crossing the blood-brain barrier to enter the brain. This data shows a water-soluble γ-cyclodextrin inclusion complex helped to deliver CRT across the blood-brain barrier. This success should fuel further pharmaceutical research on CRT in the treatment for AD, and it should engender research on γ-cyclodextrin with other drugs that have so far not been explored.

## Introduction

**Figure 1:**
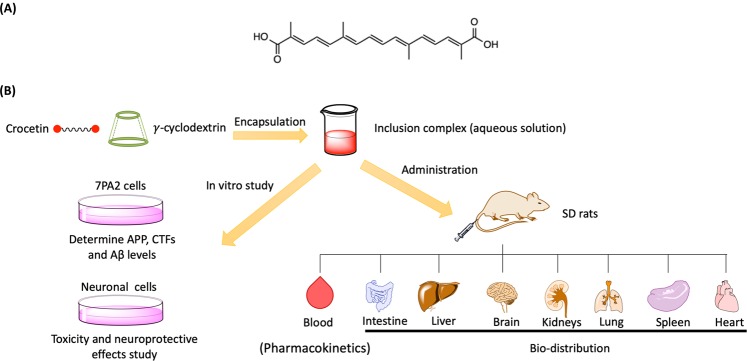
(**A**) Structure of crocetin. (**B**) Schematic representation of CRT-γ-CD for AD study.

Alzheimer’s disease (AD) is an irreversible neurodegenerative disease which cannot be cured by any therapeutic approaches up to now^1^. As the number of AD patients increases, the need to develop safe, effective drugs for AD therapy becomes increasingly urgent. In traditional herbal medicine, several plants have been used to treat the symptoms of neurodegenerative diseases^2,3^. Crocetin (CRT) is an active compound isolated from the fruits of gardenia (*Gardenia jasminoides* Ellis) and the stigmas of saffron (*Crocus sativus* L.)^4^. Various pharmacological activities of CRT have been reported. CRT can inhibit amyloid β (Aβ) fibril formation, destabilize pre-formed Aβ fibrils and improve Aβ degradation *in vitro*^5,6^. CRT can also reduce Aβ_1–42_-induced neurotoxicity by attenuating oxidative stress in murine hippocampal cells^7^. Furthermore, CRT can reduce the production of various neurotoxic molecules from neuron, such as lipopolysaccharide (LPS)-induced nitric oxide (NO), tumor necrosis factor-α (TNF-α), interleukin-1β (IL-1β), and reactive oxygen species (ROS), which provided strong protection from neuronal cell death^8,9^. Moreover, CRT has been proven to cross the blood-brain barrier (BBB) limitedly after administration^10^. The mentioned properties of CRT indicate that it may be a potentially useful candidate for AD treatment. In terms of its chemical structure (Fig. 1A), CRT contains two carboxylic acid groups at each end of a polyene chain. However, CRT is insoluble in water in the physiological range (0.0056 g/L); it can only slightly dissolve in pyridine, dimethyl sulfoxide or aqueous alkali solutions at pH above 9^11^. Poor solubility restricts the therapeutic applications of CRT. So far, due to the solubility problem, the intake of CRT is mainly via oral administration or intraperitoneal injection^9,10,12^. To maximize the therapeutic effectiveness of CRT to treat AD, a water soluble and injectable CRT formulation should be developed.

When developing a formulation to treat neurodegenerative diseases, the role of the BBB has to be considered. The BBB is the bottleneck in brain delivery as it only permits selective transport of molecules. In detail, 98% of small molecules and almost 100% of large molecules do not cross the BBB^13^. To overcome the BBB, drug delivery systems (DDS) such as cyclodextrins can be employed. Cyclodextrins (CDs) are cyclic oligosaccharides which can trap or encapsulate lipophilic molecules in the cavity to yield water soluble inclusion complex^14^. It has been reported that CDs could serve as a drug carrier to deliver and increase drug molecules across the BBB^15,16^. At the same time, CDs can also act as therapeutic agents to treat AD. CDs could reduce Aβ production and enhance Aβ clearance mechanisms in cell and animal models^17^. Based on the above rationale, encapsulating CRT into CDs should improve its solubility and bioavailability, while the unique features of CDs along with the neuroprotection properties of CRT may show promising therapeutic effects to treat AD.

According to the amyloid beta hypothesis, the pathogenesis of AD is due to the extracellular accumulation and aggregation of Aβ peptides in the brain^18^. Generally, a trans-membrane amyloid precursor protein (APP) is cleaved by β-secretase to yield C-terminus fragments CTF-α and CTF-β; the CTF-β is subsequently cleaved by γ-secretase to produce Aβ^18,19^. The accretion of Aβ peptides leads to different neurotoxic issues^18–20^. For example, Aβ has been recognized as a key factor in the neurodegeneration in AD patients and it mediates its harmful effect via inducing oxidative stress in the brain^20^. Aβ peptides and these neurotoxic issues trigger each other in a positive feedback loop^21^. Therefore, the most direct strategies to inhibit or treat AD are to decrease the generation of Aβ, increase the clearance of Aβ, and prevent Aβ aggregation formation.

In this study, a water-soluble inclusion complex involving CRT and γ-CD was successfully developed. γ-CD was chosen because γ-CD series is less toxic towards the BBB than α- and β-CD series, and γ-CD series have the largest cavity size to encapsulate CRT^22^. *In vitro* experiments were carried out to determine the therapeutic effects of CRT-γ-CD to treat AD. After that, pharmacokinetic parameters and BBB permeability of CRT-γ-CD were determined using normal SD rat models. This study provides a strategy to deliver CRT across the BBB and gives insight into further pharmaceutical research on CRT for AD treatment. It also sheds light on brain delivery of other similar drugs by employing γ-CDs.

## Results

### Characterization of CRT-γ-CD inclusion complex

#### IR Spectroscopy

**Figure 2:**
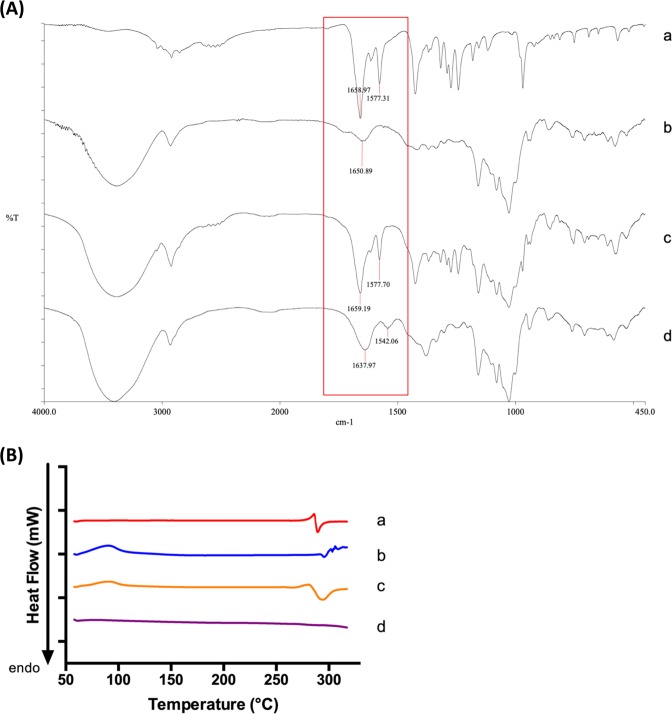
(**A**) IR spectra of (a) CRT, (b) γ-CD, (c) physical mixture and (d) inclusion complex. (**B**) DSC graph of (a) CRT, (b) γ-CD, (c) physical mixture and (d) inclusion complex.

FTIR is a very useful tool to confirm the existence of both guest and host molecules in the inclusion complex. IR spectra **(**Fig. 2A**)** of CRT, γ-CD, physical mixture of CRT and γ-CD, and CRT-γ-CD inclusion complex were obtained and compared. As seen in the figure, the spectra of the physical mixture **(c)** and the inclusion complex **(d)** differed in certain aspects. For the physical mixture, the characteristic peaks were a combination of peaks from CRT **(a)** and γ-CD single compound **(b)**. In contrast, the spectrum of the inclusion complex **(d)** looked almost identical to the spectrum of γ-CD **(b)** in the region of approximately 1200–900 cm^−1^. However, in **(d)** some of the characteristic peaks from CRT could not be seen. The missing of signature peaks of CRT indicates that the inclusion complex was successfully formed. Consistent with these spectra, the properties of physical mixture and inclusion complex were different. In the spectrum of CRT, the peak at 1658 cm^−1^ corresponds to the C=O stretching of two carboxylic groups in CRT, while the peak at 1577 cm^−1^ should be related to C=C stretching in the carbon chain of CRT. Comparing peaks in the spectrum of the inclusion complex, it can be seen that the wavenumber of the peaks was shifted. The wavenumbers of C=O stretching and of C=C stretching were shifted to 1637 and 1542 cm^−1^, respectively. The decrease in the frequency between the inclusion complex and its included molecule (CRT) is due to the changes in the microenvironment. It involves the formation of hydrogen bonding and the presence of van der Waals forces during the interaction of CRT and γ-CD to form the inclusion complex^14^. As the IR pellets were in solid form, the hydrophobic or ionic interactions between the host and guest molecules cannot be seen if there is any. Thus, the FTIR spectra provides evidence of the successful formation of the CRT-γ-CD inclusion complex.

#### Differential scanning calorimetry (DSC)

To determine the solid state of inclusion complex, DSC analysis was also performed. In Fig. 2B, it can be seen that the melting point of CRT **(a)** was determined to be 285 °C, which was close to the reference value (285–287 °C). CRT decomposed as it melted. In the graph of γ-CD **(b)**, the peak appearing at around 100 °C might be due to water molecules trapped in γ-CD, which evaporated at that temperature. The decomposition temperature of γ-CD was close to 300 °C. The graph of the physical mixture **(c)** shows the combination of peaks of CRT and γ-CD single compound. In the graph of the inclusion complex **(d)**, the melting peak of CRT has disappeared, showing that the solid was amorphous. This is further evidence that CRT has been inserted into γ-CD to form the inclusion complex.

#### Percentage yield

After confirming successful formation of the CRT-γ-CD inclusion complex, the amount of CRT in the γ-CD inclusion complex was determined by UV spectroscopy. CRT showed absorption peaks at 420 nm and 443 nm. The peak at 420 nm was chosen for quantitative analysis. The percentage yield of the inclusion complex formation was about 94.53 ± 0.01%.

### CRT formulations showed no toxicity toward neuronal cells and Alzheimer’s disease model cells

**Figure 3:**
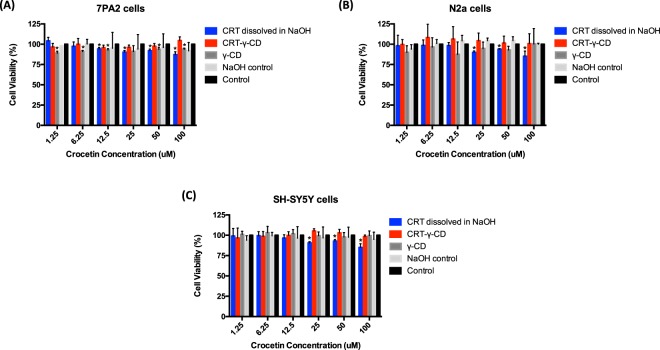
Effect of CRT formulations on cell viability in (**A**) 7PA2 cells, (**B**) N2a cells and (**C**) SH-SY5Y cells. *p < 0.05 compared with control.

The effect of CRT formulations and its components on viability of normal neuronal cells and APP overexpressed cells were evaluated by MTT assay. Normal neuroblastoma cells N2a, SH-SY5Y cells and APP overexpressed cells 7PA2 were chosen. From the results (Fig. 3), CRT-γ-CD with CRT concentrations from 1.25 to 100 μM did not show significant cytotoxic activity toward all cell lines. Viability of cell lines treated with CRT dissolved in NaOH was remained above 80%. The amount of γ-CD containing in the inclusion complex and the amount of sodium hydroxide used to dissolve CRT did not significantly affect the viability of the cells. These results suggested that both CRT and CRT-γ-CD were suitable for *in vitro* study, using these cell lines and above concentration range of CRT.

### Modulation of CTFs expression

**Figure 4:**
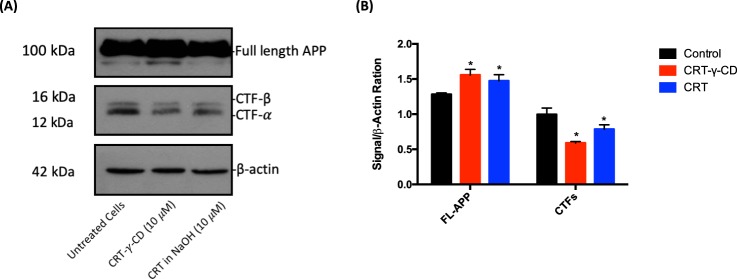
Western blot results of APP expression in 7PA2 cells after treatment with CRT formulations. (**A**) Immunoblotting detection of FL-APP and CTFs, with actin as internal standard. FL-APP and Actin were cropped from the same gel while CTFs were cropped from another gel as described in experimental procedures; (**B**) Diagrammatic presentation of quantified protein expression. *p < 0.05 compared with control; **p < 0.01 compared with control.

To evaluate the therapeutic efficacy of CRT to treat AD, effect of CRT on the levels of APP and CTFs were assessed. As shown by Western analysis in Fig. 4A, full-length APP (FL-APP) was still overexpressed in 7PA2 cells after treatment, but treatment with CRT or CRT-γ-CD could downregulate the expression of CTF-α and CTF-β in certain extend. The change of CTFs level after CRT-γ-CD treatment was greater than that of CRT. The level of CTF-α and CTF-β decreased to 76.12% ± 9.39% and 65.41% ± 9.75% in CRT-γ-CD treatment group; while 77.67% ± 12.53% and 71.48% ± 16.34% of CTF-α and CTF-β were remained after being treated with CRT, as compared to control group. This data suggests that CRT and its inclusion complex was able to decrease the level of CTF-α and CTF-β in 7PA2 cells at 10 μM.

### Modulation of Aβ level in 7PA2 cells by CRT

**Figure 5:**
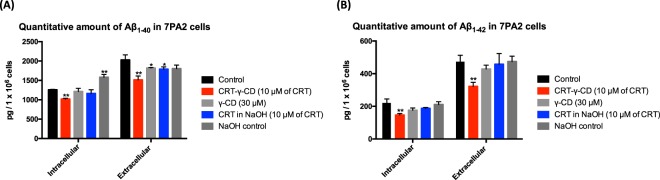
Quantitative amount of (**A**) Aβ_1–40_ and (**B**) Aβ_1–42_ in 7PA2 cells after treating with CRT formulations. *p < 0.05 compared with control; **p < 0.01 compared with control.

In amyloidogenic pathway, CTF-β is cleaved by γ-secretase to produce Aβ. As the level of CTF-β in 7PA2 cells was reduced by CRT and its inclusion complex, the levels of intracellular and extracellular Aβ were then evaluated quantitatively by ELISA assay. Fig. 5 provided evidence that CRT-γ-CD could reduce both the Aβ_1–40_ and Aβ_1–42_ levels in 7PA2 cells. After treating 7PA2 cells with 10 μM of CRT-γ-CD inclusion complex, the total amount of intracellular Aβ_1–40_ and Aβ_1–42_ dropped from to 1252.67 ± 73.73 pg to 1019.46 ± 18.06 pg (81.38 ± 1.44% of control group) and from 220.65 ± 24.42 pg to 148.56 ± 9.49 pg (66.57 ± 4.30% of control group) respectively. Extracellular levels of Aβ_1–40_ and Aβ_1–42_ were also reduced from 2030.04 ± 129.60 pg to 1514.11 ± 100.39 pg (74.59 ± 4.95% of control group) and from 471.29 ± 41.28 pg to 322.13 ± 24.97 pg (68.35 ± 5.30% of control group), respectively. When comparing the effects of CRT-γ-CD and CRT on the level of Aβ, the ability of single CRT to inhibit the production of Aβ were not as obvious as that of the inclusion complex. These findings prove that the CRT-γ-CD inclusion complex was able to downregulate CTFs expression and lower the Aβ level, which are pathological hallmarks of AD.

### Protective effect of CRT against H_2_O_2_-induced cell death

**Figure 6:**
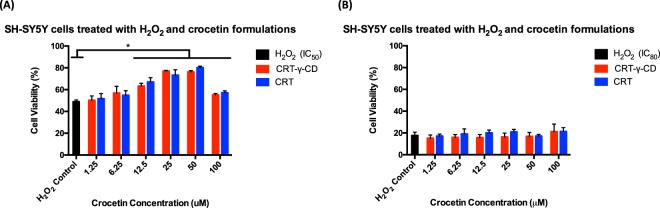
Cell viability of SH-SY5Y cells after co-incubation (**A**) 360 μM of H_2_O_2_ (IC_50_) or (**B**) 800 μM of H_2_O_2_ **(IC**_**80**_) with CRT formulations. *p < 0.05 compared with control.

Exposure of SH-SY5Y cells to various concentrations of CRT (1 to 100 μM) alone for 24 hr did not alter the viability. However, 3 hr exposure of cells to 360 μM or 800 μM of H_2_O_2_ induced significant cell death, and cell viability was almost 50% or 20% of control, respectively. Co-incubation of cells with various concentrations of CRT and 800 μM of H_2_O_2_ did not drastically affect cell viability. As demonstrated in Fig. 6, CRT dose-dependently prevented cell death due to H_2_O_2_ treatment from CRT concentrations of 12.5 μM to 50 μM when the concentration of H_2_O_2_ was 360 μM. The cell viability was improved from 48.93 ± 1.59% to 77.01 ± 0.58% when cells were treated with 50 μM of CRT-γ-CD. There was no significant difference between CRT-γ-CD and the CRT treatment group. These results showed the effectiveness of CRT in preventing H_2_O_2_-induced toxicity to neuronal cells.

### Pharmacokinetics study of CRT

**Figure 7:**
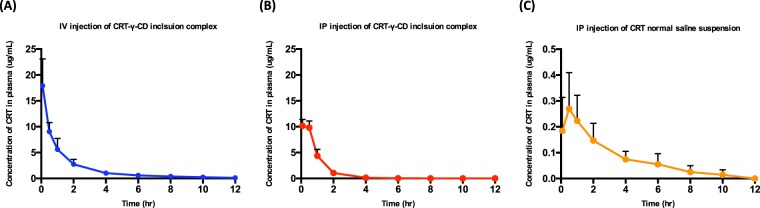
Concentration-time curve of CRT pharmacokinetic profile in SD rats (n = 6) via (**A**) IV injection of CRT-γ-CD inclusion complex; (**B**) IP injection of CRT-γ-CD inclusion complex; (**C**) IP injection of CRT normal saline suspension.

CRT concentrations in plasma at different time points were obtained. The average drug concentration-time curves of CRT in SD rats are shown in Fig. 7. The main pharmacokinetic parameters are shown in Table 1. From the table, it can be seen that CRT concentration in plasma peaked immediately after intravenous injection. The peak concentration (C_max_) was 17.903 ± 4.772 μg/mL. Intravenous injection of CRT-γ-CD inclusion complex had the largest area under the curve (AUC_0-∞_), which was 21.991 ± 4.613 μg·h^−1^·mL^−1^. For intraperitoneal injection of CRT-γ-CD inclusion complex and CRT normal saline suspension, both the T_max_ were at around 0.5 hr. However, there were large differences for C_max_ and AUC_0-∞_. C_max_ of inclusion complex solution was 10.528 ± 2.358 μg/mL while it was 0.272 ± 0.052 μg/mL for CRT suspension, and the area under the curve (AUC_0-∞_) was determined to be 11.767 ± 2.138 μg·h^−1^·mL^−1^ and 0.963 ± 0.278 μg·h^−1^·m L^−1^, indicating that the absorption of CRT-γ-CD in SD rats was much greater than that of CRT normal saline suspension. From above results, it can be demonstrated that the bioavailability of CRT was greatly improved when it was administrated in inclusion complex form.

**Table 1:** Pharmacokinetic parameters of CRT delivered via different administration routes in SD rat (n = 6).

PK parameters	IV injection of CRT-γ-CD inclusion complex	IP injection of CRT-γ-CD inclusion complex	IP injection of CRT normal saline suspension
T_1/2_ (h)	2.663 ± 1.346	0.679 ± 0.162**	2.730 ± 0.752
T_max_ (h)	0.083 ± 0.000**	0.292 ± 0.228	0.500 ± 0.000
C_max_ (μg/mL)	17.903 ± 5.188**	10.460 ± 1.172**	0.240 ± 0.144
AUC_0-t_ (μg·h^−1^·mL^−1^)	21.461 ± 4.459**	12.169 ± 1.387**	0.801 ± 0.434
AUC_0-_ ∞ (μg·h^−1^·mL^−1^)	22.105 ± 4.751**	12.211 ± 1.378**	0.935 ± 0.441

(T_1/2_: half-life; T_max_: time to reach highest plasma concentration; C_max_: peak plasma concentration; AUC: Area under the curve; *p < 0.05 compared with suspension; **p < 0.01 compared with suspension).

### Bio-distribution of CRT

**Figure 8:**
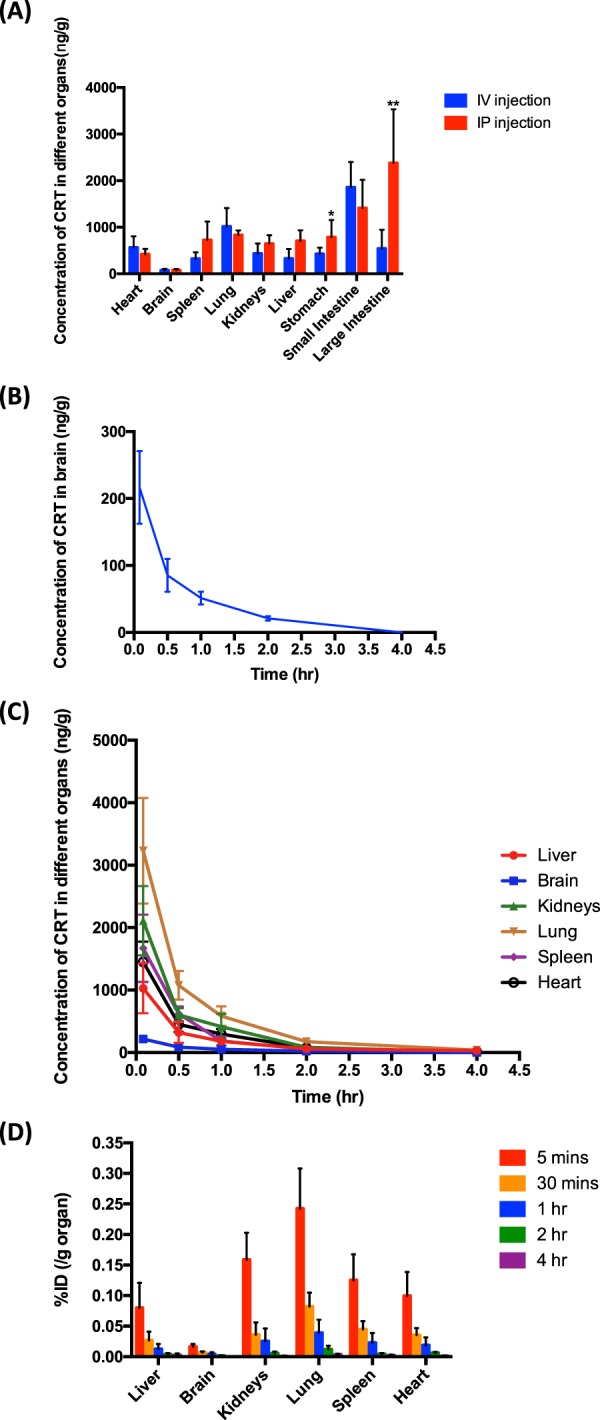
Bio-distribution profiles of CRT in SD rats (n = 5). (**A**) CRT distribution profiles in different tissues after IV or IP administration for 0.5 hr. *p < 0.05; **p < 0.01. (**B**) Concentration-time curve of CRT distributed in the brain of SD rats after IV administration. (**C**) Concentration-time curve of CRT distributed in different tissues after IV administration. (**D**) Percentage of injected dose (%ID) per g tissue of CRT after IV administration.

By comparing the CRT distribution in organs via intravenous injection and intraperitoneal injection of CRT-γ-CD inclusion complex after 0.5 hr (Fig. 8A), the amount of CRT in different organs showed no significant difference between the two administration routes, except in the large intestine and stomach.

CRT could cross the blood-brain barrier and enter the brain after injection by both methods. According to the pharmacokinetics results in **2.6**, intravenous injection showed greater bioavailability than intraperitoneal administration. Therefore, the distribution profiles of CRT in main organs via intravenous injection of CRT-γ-CD inclusion complex were further studied. Fig. 8C and Fig. 8D showed the distribution of CRT in various tissues at five dosing points. It was found that the concentration of the drug in each tissue reached its maximum at 0.833 h (5 mins). Percentage injected dose (%ID) in per gram of brain was determined to be 0.016 ± 0.004%. There was almost no drug residue after administration for 4 hr. Together, these results indicate that CRT could distribute to various tissues in the body including the brain immediately, and was subsequently metabolized relatively quickly.

## Discussion

To our knowledge, this is the first report of studies employing γ-CD as a drug delivery system to improve the solubility and bioavailability of CRT. Previously, similar phenomena have been reported by others using other substances with CDs, and the results have been reviewed^23^. This study developed a water-soluble inclusion complex consisting of CRT and γ-CD, which greatly overcame the solubility problem of CRT. By using our preparation methodology, the encapsulation efficiency of inclusion complex was over 95%. It seems to be cost-effective and feasible for large-scale industrial production in future. Moreover, preparation of the inclusion complex did not involve the use of organic solvent, thereby avoiding the toxicity typically associated with such solvents. The safeness of the active pharmaceutical ingredient, excipient and inclusion complex were evaluated. In the toxicity study, it was proven that CRT, γ-CD, and the CRT-γ-CD inclusion complex were not toxic to neuronal cells nor to AD model cells.

To evaluate the therapeutic efficiency of CRT formulations, *in vitro* study firstly focused on their ability to regulate the expression of some proteins in AD model cells. CRT and CRT-γ-CD inclusion complex could significantly downregulate the expression of CTFs as compared to the control group. It can be explained by other study that CRT could downregulate the expression of β-secretase (BACE1), which is responsible for the cleavage of the FL-APP and the production of CTFs^24^. Due to the decrease of CTFs, it will affect the production of Aβ. ELISA assay was performed to qualitatively determine the amount of Aβ_1–40_ and Aβ_1–42_. In our study, CRT-γ-CD inclusion complex could significantly decrease both intracellular and extracellular levels of Aβ. These decreases can be explained by previous observations^24^. The production of Aβ from CTFs is regulated by γ-secretase which includes PSEN1 and PSEN2 as catalytic components. CRT, apparently, significantly suppressed the levels of both PSEN1 and PSEN2^24^. At the same time, our results are in agreement with previous works in which CRT was shown to inhibit the formation of Aβ fibrils and promote Aβ degradation^5,6^. These findings indicate that the reduction of intracellular Aβ levels should be related to the increase of CRT-induced Aβ degradation^6^. Similar to another report, CRT-γ-CD inclusion complex also achieved its therapeutics effects without affecting cell viability^25^. Moreover, molecules with similar structure to CRT were also found to inhibit Aβ aggregation^26^. Therefore, it can be assumed that CRT-γ-CD inclusion may be a potential formulation for AD therapy. It is notable that the therapeutic effect of CRT-γ-CD inclusion complex was stronger than that of CRT. It can be explained by following reason: CRT was dissolved by 0.1 M of NaOH to yield a 1 mg/mL solution. After diluting the drug solution with medium and incubating it for 24 hr, the pH of the medium was decreased because CO_2_ in the atmosphere of the incubator dissolved in the medium to form carboxylic acid and neutralize the NaOH. As a result, CRT would precipitate from the solution. It would weaken the effect of CRT to treat AD when placing CRT solution in the incubator for long time.

Because oxidative stress and ROS also contribute to the pathology of AD, the antioxidant activities of CRT and CRT-γ-CD inclusion complex were also evaluated^27–29^. Former studies found that CRT was able to protect mouse hippocampal-derived Ht22 cells from Aβ_1–42_-induced neuronal cell death and reduce ROS formation cause by Aβ_1–42_^7^. CRT could also protect SH-SY5Y cells against cellular apoptosis by reducing ROS production induced by H_2_O_2_ and decreasing caspase-3 activation^9^. Similar protective effects of saffron and CRT by altering antioxidant enzymatic activities have also been observed in other cell lines and animal models^30–33^. Our results showed that both CRT and CRT-γ-CD inclusion complex could protect SH-SY5Y cells from H_2_O_2_-induced neurotoxicity in a dose-dependent manner when SH-SY5Y cells were treated with 360 μM of H_2_O_2_ (IC_50_). Furthermore, previous study reported that the antioxidative activities of CRT are due to the double bonds and methyl groups on its polyene chain^34^. This provides additional information about the structure and formation of CRT-γ-CD inclusion complex. The interactions of CRT and γ-CD mainly happen between the carboxylic groups of CRT and the hydroxyl groups of γ-CD.

In pharmacokinetics study, the pH of the formulation must be adjusted to the physiological range in order to be suitable for intravenous injection. During the preparation of the inclusion complex, sodium chloride was produced as byproduct when HCl was used to neutralize the NaOH present in the solution (from pH 12.5 to pH 7.4). The sodium chloride was not further removed from the inclusion complex solutions as its percentage was less than 0.9% in solution. In this situation, the ion concentration or osmotic pressure of solution would not be too high for intravenous injection. In our study, intravenous injection of CRT-γ-CD inclusion complex showed better absorption than intraperitoneal injection of CRT formulations. In previous reports, when a micelle solution containing CRT (40 nmol) was orally administered to mice, CRT was absorbed into the blood circulation rapidly and reached its highest plasma concentration (49 ng/mL) at about 0.5 hr^12^. Sonali *et al*. determined highest plasma concentration of CRT (about 10 μg/mL) to occur approximately 2 hr after oral administration of a CRT dose of 50 mg/kg. Data of intravenous administration was also provided; however, the method for preparing the CRT solution was not described^35^. CRT has also been orally administered to humans. The subjects received CRT or saffron tea, and the maximum CRT concentration was observed after 2 hr to 4.8 hr, the concentrations of CRT detected in the blood were ranging from 100.9 to 279.7 ng/mL^36,37^. Compared to previous results, either intravenous administration or intraperitoneal injection of CRT-γ-CD inclusion complex could significantly reduce the dose of CRT to achieve a higher plasma concentration, and shorten the time to reach peak concentration.

In biodistribution study, the distribution profiles of CRT-γ-CD inclusion complex in tissues 0.5 hr after intravenous injection and intraperitoneal injection were compared. The concentration of CRT in most of the tissues basically did not significantly differ, comparing the two administration routes. However, for intravenous injection, CRT levels in large intestine and stomach were significantly less than those of intraperitoneal injection. A possible reason was that CRT was absorbed via intestinal cells through a passive diffusion during intraperitoneal injection^38^. Therefore, it was quickly distributed to the stomach, large intestine and small intestine. For intravenous injection, the amount of CRT determined in the small intestine was higher than that of intraperitoneal injection. One possible reason, as suggested by a previous report, is that CRT is partly metabolized in the intestinal mucosa^12^.

CRT can bind to albumin in blood plasma by occupying fatty acid binding sites so that CRT can be distributed into different tissues^39,40^. CRT was reported to be able to penetrate the BBB *in vitro* and *in vivo*^41,42^. CRT could serve as a substrate for pGP efflux pump and penetrate the BBB slowly by using the transcellular diffusion pathway^41^. After oral administration of CRT at 100 mg/kg for 90 minutes, about 2.43 nmol/g (equal to 798 ng/g) of CRT was determined in the brain^42^. In our study, CRT was also detected in the brain; the highest concentration of CRT was determined after administration (5 mg/kg) for half hour, which was about 200 ng/g of brain. Our results proved that the used of γ-CD was able to facilitate the CRT across the BBB and reach the brain, with a higher efficiency (approximately 5 times) than previous report^42^.

## Conclusion

In this study, we developed an effective method to prepare CRT-γ-CD inclusion complex that enhances the solubility, bioavailability and applicability of CRT. This is the first report of employing γ-CD to deliver CRT across the BBB. Therapeutic efficacy and safety of the inclusion complex were elucidated. The complex did not show significant toxicities to normal neuronal cells or APP overexpressed cells. It could downregulate the expression of CTFs and reduce Aβ production in AD model cells. It also showed neuroprotective and antioxidant effects against H_2_O_2_-induced cell death. In animal study, it was proven that the developed formulation was suitable for injection. After injection, CRT penetrated the BBB and localized inside the brain. These results provide some meaningful information for pharmaceutical research on CRT for AD therapy, and provide hints for the general development of γ-CD-based drug delivery systems by modifying the chemical structure of γ-CD to deliver other drugs across the BBB with higher efficiency for AD treatment.

## Materials and Methods

### Materials

Crocetin was obtained from Jinan University (Guangzhou, China). Heparin sodium, dimethyl sulfoxide, sodium hydroxide, sodium chloride, γ-CD, hydrochloric acid and hydrogen peroxide were purchased from Sigma-Aldrich (Saint Louis, MO, USA). 3-(4,5-dimethylthiazol-2-yl)-2,5-diphenyltetrazolium bromide (MTT) was purchased from Invitrogen (Waltham, MA, USA). Methanol was purchased from VWR Chemicals (Leicestershire, UK). All chemicals and reagents were of analytical grade.

### Cell cultures

Mouse neuroblastoma N2a cells and Chinese hamster ovary cells stably transfected with human APP751 bearing the V717F mutation (7PA2 cells) were cultured in Dulbecco’s modified Eagle’s medium (DMEM) with GlutaMAX supplemented with 10% fetal bovine serum, 100 U/mL penicillin, and 100 μg/mL streptomycin (Thermo Fisher Scientific, Waltham, USA) (and 200 μg/mL G418 for 7PA2 cells) at 37 °C in a humidified incubator with a 5% CO_2_ atmosphere. Human neuroblastoma SH-SY5Y cells were cultured in DMEM/F12 (Thermo Fisher Scientific, Waltham, USA) with 10% fetal bovine serum, 100 U/mL penicillin, and 100 μg/mL streptomycin at 37 °C in a humidified incubator with a 5% CO_2_ atmosphere.

### Animals

SD rats were purchased from the Laboratory Animal Services Centre, The Chinese University of Hong Kong and acclimatized for 7 days after arrival.

### Experimental procedures

All experimental protocols and procedures of animal study were approved and done according to the guidelines of the Committee on the Use of Human & Animal Subjects in Teaching & Research of Hong Kong Baptist University and the Health Department of the Hong Kong Special Administrative Region.

#### Preparation of CRT-γ-CD inclusion complex

First, 5.0 mg of CRT was dissolved in 0.5 mL of 0.1 M sodium hydroxide solution and 59.2 mg of γ-CD was dissolved in 0.2 mL of purified water (Molar ratio of CRT to γ-CD was 1:3). Next, the CRT solution was added dropwise to the aqueous γ-CD solution. The mixed solution was treated by ultrasonic homogenizer (Model 3000, Biologics Inc., NC, USA) for 4 hr for encapsulation. After the reaction was completed, the solution was neutralized by 0.1 M hydrochloric acid, and the pH of solution was adjusted to 7.4. Then the solution emulsion was extruded through a 0.2 μm pore-sized polycarbonate membrane (Millipore Co., Bedford, MA, USA). CRT concentration in solution was determined by UV spectrophotometer (Agilent, Santa Clara, CA, USA) at 420 nm. Encapsulation efficiency of CRT was calculated from the following formula: (Encapsulated CRT/Total CRT added) ×100%.

#### Characterization of CRT-γ-CD inclusion complex

Successful preparation of the CRT-γ-CD inclusion complex was confirmed by infrared spectrometer and differential scanning calorimetry (PerkinElmer, Waltham, MA, USA) as previous described^43^. Briefly, for IR analysis, the pellets were prepared by grinding 1 to 2 mg of sample and 200 mg of potassium bromide by using a pestle. Fourier Transform Infrared Spectroscopy (FTIR) spectra of the samples were obtained in the range of 450–4000 cm^−1^. The resolution was 4 cm^−1^. For DSC analysis, about 5 mg of samples were weighed and placed in aluminum pans with pinhole lids, followed by heating at a rate of 10 °C/min in the temperature range of 60 °C to 320 °C. The measurements were carried out under dry nitrogen at the flow rate of 50 mL/min. An empty aluminum pan was used as reference.

#### Cytotoxicity studies of CRT formulations

The cytotoxicity of free CRT (dissolved in 0.1 M NaOH solution at 1 mg/mL) and CRT-γ-CD inclusion complex (aqueous solution) against SH-SY5Y, N2a and 7PA2 cells was evaluated by MTT assay. SH-SY5Y, N2a and 7PA2 cells were seeded onto 96-well plates with 1 × 10^4^ cells per well. After 24 hr incubation, the cell culture medium was replaced with fresh medium containing different formulations at CRT concentrations ranging from 1 to 100 μM, and cells were incubated for another 24 hr. Then 20 μL of MTT solution (5 mg/mL in PBS) was added to each well, and cells were further incubated for 4 hr at 37 °C. Then the medium was removed and 100 μL of DMSO was added to each well to dissolve the formazan crystals formed by the living cells. Untreated cells in complete cell culture medium were used as control. The absorbance was measured by Benchmark Plus Microplate Reader at 570 nm. Cell viability (%) was calculated as A_treated_/A_control_ × 100%, where, A_treated_ and A_control_ represented the absorbance of cells treated with different formulations and blank culture medium, respectively.

#### Western blot analysis

7PA2 cells were seeded onto 6-well plates with 1 × 10^6^ cells per well in full DMEM medium. After 24 hr of incubation, the cell culture medium was replaced with DMEM medium containing different formulations at CRT concentration of 10 μM, and cells were incubated for another 24 hr. Control (γ-CD or NaOH control) and untreated cultures were tested in parallel. For cell homogenate preparation, cells were washed with phosphate buffer solution (PBS) and solubilized in ice-cold RIPA buffer (20 mM Tris, pH 7.4, 150 mM NaCl, 1% triton-X, 0.5% sodium deoxycholate, EDTA), protease inhibitor cocktails (EMD chemical Inc, Gibbstown, NJ, USA). 30 μg of the cell lysates was separated on 10% SDS-PAGE gel for detection of full-length APP and β-actin or 50 μg of the cell lysates was separated on 15% SDS-PAGE gel for detection of CTF-𝛼 and CTF-β, respectively. Then, the proteins were transferred to PVDF membrane (GE Healthcare, Piscataway, NJ, USA). After blocking with 5% skim milk, the blotted membranes were probed overnight at 4 °C. Primary antibodies used for Western blot included: Anti-beta amyloid polyclonal CT695 (which recognizes the carboxyl terminus of APP and CTFs; 1:3000); β-actin (as an internal reference control, 1:3000). Antibodies were purchased from Thermo Fisher Scientific (Waltham, USA).

#### Determination of Aβ_1–40_ and Aβ_1–42_ level by ELISA

The levels of extracellular and intracellular of Aβ_1–40_ and Aβ_1–42_ were determined by using ELISA assay kit (Genetimes Technology Inc., Shanghai, China). Briefly, 7PA2 cells were seeded onto 6-well plates with 1 × 10^6^ cells per well in serum-free DMEM medium. After 24 hr of incubation, the cell culture medium was replaced with serum-free DMEM medium containing different formulations at CRT concentration 10 μM, and cells were incubated for another 24 hr. Control (γ-CD or NaOH control) and untreated cultures were tested in parallel. Then conditioned media were collected for ELISA analysis to measure the extracellular Aβ_1–40_ and Aβ_1–42_.

Cells were lysed for intracellular Aβ_1–40_ and Aβ_1–42_ determination, as previously reported with slight modifications^44^. Cells were harvested using 0.5 mL of 0.25% trypsin. An equal volume of full DMEM medium was added to quench further reaction. Then cell solution was centrifuged at 1000 rpm for 5 min. The supernatant was discarded. Then pellets were resuspended in 0.5 mL of PBS twice for washing. 100 μL of 1% SDS was added to the cell pellets to lyse the cells. After being briefly sonicated, the clear solution was centrifuged at 15,000 rpm at 4 °C for 15 minutes. Supernatant was collected and diluted with PBS 10 times before ELISA assay.

#### Protective effect of crocetin against H_2_O_2_-induced cell death

SH-SY5Y cells were seeded onto 96-well plates with 1 × 10^4^ cells per well. After 48 hr incubation, the cell culture medium was replaced with fresh medium containing different formulations at CRT concentrations ranging from 2 to 200 μM (100 μL) and fresh medium containing 720 μM or 1600 μM of H_2_O_2_ (100 μL), and cells were incubated for another 3 hr. Then 20 μL of MTT solution (5 mg/mL in PBS) was added to each well and cells were further incubated for 4 hr at 37 °C. Then the medium was removed and 100 μL of DMSO was added to each well to dissolve the formazan crystals formed by the living cells. Untreated cells in complete cell culture medium were used as control. The absorbance was measured by Benchmark Plus Microplate Reader at 570 nm. Cell viability (%) was calculated as A_treated_/A_control_ × 100%, where A_treated_ and A_control_ represented the absorbance of cells treated with different formulations and blank culture medium, respectively.

#### Pharmacokinetics study of CRT

18 healthy SD rats weighing about 400 g were divided into 3 groups randomly. CRT-γ-CD inclusion complex was administered via intravenous injection at the tail vein or intraperitoneal injection with a dose of 5 mg of CRT/kg body weight. CRT normal saline suspension was administered via intraperitoneal injection with a dose of 5 mg of CRT/kg body weight. 0.3 mL of blood samples were collected from the tail of the rats at predetermined time points (0.083, 0.25, 0.5, 1, 2, 4, 6, 8, 10, 12, 24 hr). 0.1% of heparin solution was used to rinse the sample collectors to prevent blood clotting. The blood samples were centrifuged immediately at 15,000 rpm at 4 °C for 15 minutes. Supernatant layer of plasma was then separated and stored at −20 °C until analysis. The rats were kept awake in this PK study, and water was provided during the blood collection process in order to complement water after blood loss. CRT in plasma was extracted with dimethyl sulfoxide/methanol co-solvent (1:4 V/V) system and the concentration of CRT was determined by ultra-performance liquid chromatography (UPLC, ACQUITY UPLC System, Waters, Milford, MA) using an ACQUITY UPLC BEH Shield RP 18 column (1.7 μm, 2.1 mm × 100 mm, Waters) with detection wavelength at 420 nm. The injection volume was 2 μL. Column temperature was 40 °C. Mobile phase was water with 0.1% formic acid and acetonitrile with 0.1% formic acid in ratio (V:V) from 35:65 to 5:95 (0 to 7 mins); 5:95 to 35:65 (7 to 8 mins); 35:65 (8 to 10 mins). The flow rate was 0.3 mL/min. The data were analyzed using pharmacokinetic software PKsolver, and the pharmacokinetic parameters were calculated using the non-compartment model^45^.

#### Bio-distribution study of CRT

30 healthy SD rats weighing about 400 g were divided into 6 groups randomly. CRT-γ-CD inclusion complex was administered into 5 groups of rats via intravenous injection at the tail vein and 1 group of rats via intraperitoneal injection at a dose of 5 mg of CRT/kg body weight. 4 groups of rats administered via intravenous injection were sacrificed at each predetermined time points (0.0833, 1, 2 or 4 hr after administration) to collect the brain, heart, lung, liver, kidneys and spleen. 10 rats in the remaining 2 groups were sacrificed after administration for 0.5 hr to collect brain, heart, lung, liver, kidneys, spleen, intestine and stomach. The tissues were ground to obtain tissue homogenates by using an Ultra-Turrax disperser (IKA, Germany). CRT in homogenates was extracted with a dimethyl sulfoxide/methanol co-solvent (1:4 V/V) system. 1 mL of solvent was added to 1 g of organ homogenate for extraction. Blood was also collected to determine the concentration of CRT in plasma. The CRT concentration in plasma and tissue homogenates was monitored by UPLC as above described. Percentage injected dose per g of each organ were calculated according to the following formula: 100% x (Amount of CRT detected in organ/Actual CRT injected).

#### Statistics analysis

All data are presented as mean ± SD unless specified otherwise. All statistical analyses were performed using GraphPad Prism 6.0 software. The statistical significance of the data was assessed using Student’s t-tests or one-way analysis of variance. A p < 0.05 was considered to be significant (denoted by *), and a p < 0.01 was considered as highly significant (denoted by **).
